# A Case Report of Recurrent Takotsubo Cardiomyopathy in a Patient during Myasthenia Crisis

**DOI:** 10.1155/2017/5702075

**Published:** 2017-10-19

**Authors:** Anusha Battineni, Naresh Mullaguri, Shail Thanki, Anand Chockalingam, Raghav Govindarajan

**Affiliations:** ^1^Department of Neurology, University of Missouri, 1 Hospital Drive, Columbia, MO 65212, USA; ^2^Department of Cardiology, University of Missouri, 1 Hospital Drive, Columbia, MO 65212, USA

## Abstract

**Introduction:**

Patients with myasthenia crisis can develop Takotsubo stress cardiomyopathy (SC) due to emotional or physical stress and high level of circulating catecholamines. We report a patient who developed recurrent Takotsubo cardiomyopathy during myasthenia crisis. Coexisting autoimmune disorders known to precipitate stress cardiomyopathy like Grave's disease need to be evaluated.

**Case Report:**

A 69-year-old female with seropositive myasthenia gravis (MG), Grave's disease, and coronary artery disease on monthly infusion of intravenous immunoglobulin (IVIG), prednisone, pyridostigmine, and methimazole presented with shortness of breath and chest pain. Electrocardiogram (ECG) showed ST elevation in anterolateral leads with troponemia. Coronary angiogram was unremarkable for occlusive coronary disease with left ventriculogram showing reduced wall motion with apical and mid left ventricle (LV) hypokinesis suggestive of Takotsubo stress cardiomyopathy. Her symptoms were attributed to MG crisis. Her symptoms, ECG, and echocardiographic findings resolved after five cycles of plasma exchange (PLEX). She had another similar episode one year later during myasthenia crisis with subsequent resolution in 10 days after PLEX.

**Conclusion:**

Takotsubo cardiomyopathy can be one of the manifestations of myasthenia crisis with or without coexisting Grave's disease. These patients might benefit from meticulous fluid status and cardiac monitoring while administering rescue treatments like IVIG and PLEX.

## 1. Introduction

Myasthenia gravis (MG) is a progressive autoimmune neuromuscular junction disorder that affects skeletal muscle leading to considerable morbidity and rarely mortality. Myasthenia crisis is defined as acute worsening of weakness in bulbar and respiratory muscles requiring mechanical ventilation [1]. The primary mode of treatment is immunomodulation with high-dose oral steroids, intravenous immunoglobulin (IVIG), and plasma exchange (PLEX) to clear the acetylcholine receptor autoimmune antibodies, which are responsible for neuromuscular junction dysfunction [1]. Myasthenia patients may have other coexisting autoimmune diseases like thyroiditis and polymyositis which can impact their disease course and treatment [2]. Myasthenia crisis patients may have high levels of circulating catecholamines due to physical and emotional stress, which can cause stress-induced cardiomyopathy [3–6]. There have been several case reports of patients developing Takotsubo stress cardiomyopathy (SC) with left ventricular (LV) dysfunction during myasthenia crisis but its recurrence is rare [7]. We are reporting a patient with seropositive MG and Grave's disease who developed different variants of SC one year apart during subsequent myasthenia crisis posing a significant challenge to administering immunomodulatory treatments.

## 2. Case Report

**Figure 1:**
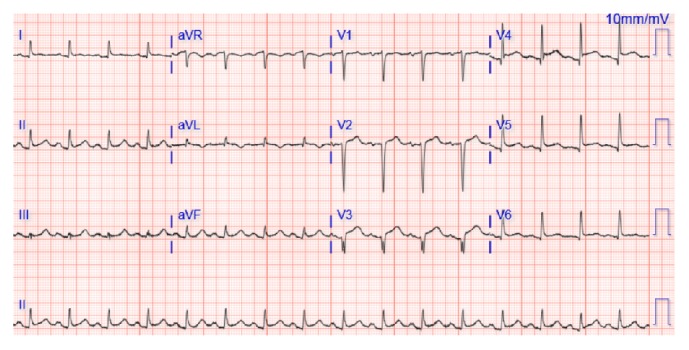
ECG during first episode of Takotsubo stress cardiomyopathy with ST elevation V1–V6, Q waves in V1–V3.

**Figure 2:**
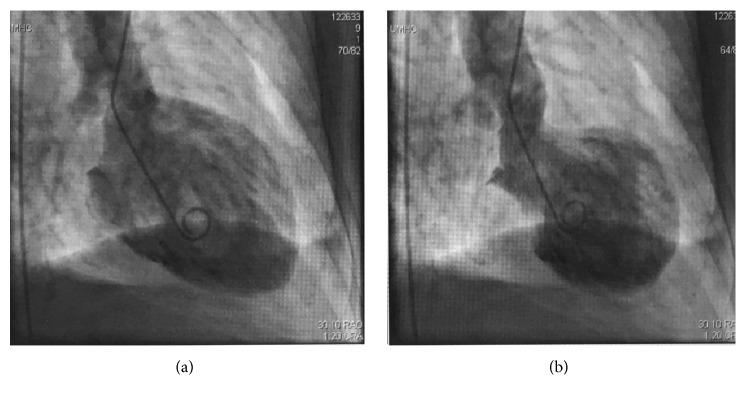
Left ventriculogram in RAO projection in diastole (a) demonstrates normal cavity contour and in systole (b) preserved basal and apical contractility with akinesia of the mid ventricle consistent with Takotsubo stress cardiomyopathy.

**Figure 3:**
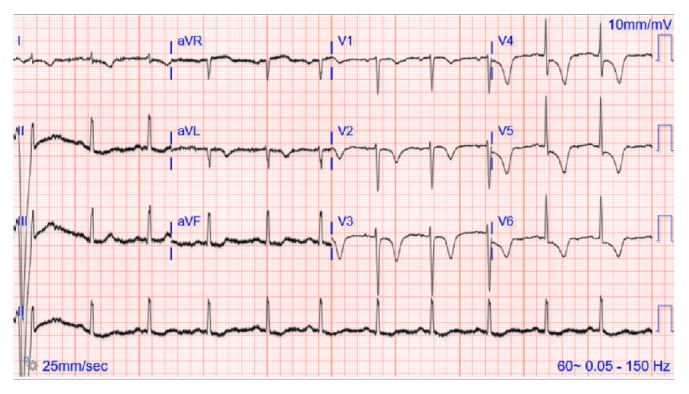
ECG during second SC episode with deep symmetrical T inversions in leads V1–V6.

**Figure 4:**
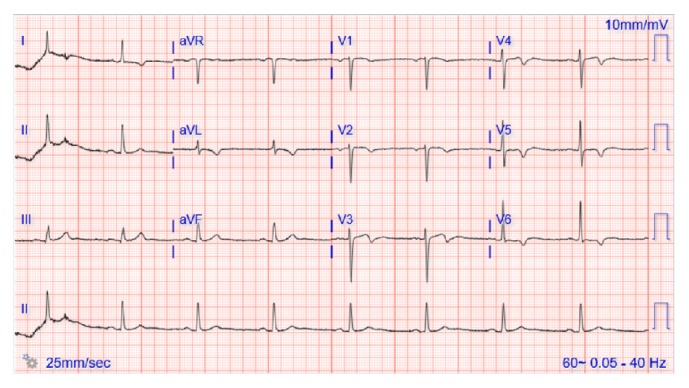
ECG 3 days later showing nonspecific ST-T wave changes only.

A 69-year-old Caucasian female with seropositive myasthenia gravis on monthly maintenance intravenous immunoglobulin (1 gram/kg body weight) every 4 weeks and prednisone 50 mg PO daily and pyridostigmine 60 mg three times a day was transferred to the university hospital cardiology service with acute onset chest pain, palpitations, nausea, and profuse sweating on waking up in the morning at a long-term acute care (LTAC) facility. The symptoms did not relieve with multiple doses of sublingual nitrate. Her medical history is significant for hypertension, coronary artery disease status after myocardial infarction one year earlier with stent placement in left anterior descending artery (LAD), chronic obstructive pulmonary disease with a recent exacerbation with respiratory failure requiring tracheostomy, Grave's ophthalmopathy on oral methimazole 10 mg daily, and 2 packs per day smoking history. She also complained of worsening double vision, slurred speech with nasal quality, difficulty with swallowing her own saliva, and generalized weakness. Her physical examination revealed severe ophthalmoparesis in horizontal and vertical directions, worsening bilateral proptosis, facial diplegia with drooling, and nasal intonation. Wheezing was noted in the upper lobes as well as crepitations in bilateral basal regions. Cardiovascular examination revealed S3 gallop, no raised jugular venous pressure, and regular pulses. Neurological exam was consistent with generalized weakness in proximal and distal muscles with easy fatigability and absent deep tendon reflexes. Her initial ECG showed ST elevations in the anteroseptal leads (V1–V4) (Figure 1) and troponin elevation concerning for ST elevation myocardial infarction (STEMI) and she had emergent cardiac catheterization. No new coronary lesions were detected and prior stent was patent. Left ventriculogram suggested severe LV dysfunction and transthoracic echocardiogram (TTE) confirmed mid ventricular variant Takotsubo stress cardiomyopathy with reduced ejection fraction (EF) of 25% (Figure 2 and Table 1). Her TSH was elevated to 7.6 from 2.3 micro unit/ml with elevated anti-thyroglobulin and anti-thyroid peroxidase (TPO) autoantibodies suggesting worsening of Grave's disease for which endocrinology recommended continuing high-dose oral steroids (Table 1). Neurology team was consulted for myasthenia crisis management, and they recommended PLEX and opined that the crisis was probably secondary to Grave's disease and methimazole that was started about 3 weeks earlier. She was managed conservatively with resolution of all her symptoms, ECG findings, and troponemia with 5 cycles of PLEX and was discharged back to the LTAC. The repeat TTE 4 weeks from the day of admission showed complete resolution of left ventricular dysfunction. She was continued on oral prednisone 50 mg daily and monthly IVIG (1 gram/kg body weight). One year later, she had a similar episode of ophthalmoparesis, generalized weakness with acute chest pain, troponemia with deep T wave inversions in V1–V6 leads on ECG (Figure 3) with TTE showing mid and apical akinesia, and EF 30% consistent with classical apical ballooning type of Takotsubo stress cardiomyopathy (Table 1). Her Grave's disease was well controlled with normal TPO autoantibodies and TSH (Table 1). We diagnosed her with recurrent Takotsubo stress-induced cardiomyopathy secondary to myasthenia crisis and treated her with 5 cycles of PLEX with complete resolution of the ST elevations (Figure 4) and troponemia and she was discharged to rehabilitation facility. Repeat TTE 3 weeks later showed complete normalization of LV function and wall motion (EF 60%).

## 3. Discussion

Myasthenia gravis is an autoimmune disorder of the neuromuscular junction predominantly in the skeletal muscles. Patients with this disease develop myasthenia crisis due to various reasons like infection, medications, and coexisting autoimmune diseases [1]. This condition and its management add significant amount of physical and psychological stress predisposing patients towards Takotsubo or stress cardiomyopathy [2–7]. Stress cardiomyopathy is a well-known complication associated with neurointensive care patients with subarachnoid hemorrhage, stroke, traumatic brain injury, and status epilepticus [8]. Rarely, certain neuromuscular junction disorders like myasthenia crisis also precipitate SC probably secondary to increased levels of circulating catecholamines [3–6, 9]. The commonest form is apical ballooning syndrome characterized by apical and mid wall akinesis and hypercontractile basal left ventricular segments [10]. Mid and basal LV segments or focal variants are also increasingly recognized now [10]. Coexistence of myasthenia gravis and Grave's disease in patients is well known and sometimes difficult to manage as treatment of one disease can worsen the other like in our patient [2, 11–17]. Three weeks prior to her first episode of SC, she had worsening of her Grave's disease as manifested by proptosis and rising levels of TPO autoantibodies for which methimazole (10 mg daily) was started. Methimazole can unmask an underlying neuromuscular junction disorder in patient with Grave's disease or trigger myasthenia crisis in patients with coexisting myasthenia gravis [18]. Both Grave's disease and myasthenia crisis were independently known to cause Takotsubo cardiomyopathy [19, 20]. In our patient, the combination of hyperthyroidism and methimazole might have triggered myasthenia crisis and subsequently SC. Over a dozen case reports describe Takotsubo cardiomyopathy complicating hyperthyroidism [19]. Coexisting coronary artery disease could cause chronic LV dysfunction with remodeling/thinning or new wall motion changes with ischemia. These factors add to the complexity, but SC can be diagnosed confidently in many instances based on carefully evaluating and recognizing SC-induced characteristic wall motion abnormalities [8, 10]. LV dysfunction improved after both diseases were managed appropriately but required prolonged hospitalization. She was continued on maintenance monthly IVIG infusions (1 gram/kg body weight) and oral prednisone (50 mg daily) and, one year later, she developed subsequent crisis and recurrence of SC. We did not find any significant trigger for her crisis after an extensive evaluation and her symptoms resolved completely with PLEX in 10 days. We think her subsequent episode of SC resolved quickly compared to the first episode as her Grave's disease was well controlled. We propose that MG patients in crisis should be screened for other autoimmune disorders like Grave's disease, which can affect neuromuscular junction and cardiac muscle. Neuromuscular specialists and intensivists managing myasthenia crisis patients should be aware of SC. If patient's cardiac symptoms like chest pain, troponemia, or ischemic ECG change during crisis, early recognition of SC and coronary artery disease can improve prognosis [20]. IVIG is contraindicated in patients with low ejection fraction as it may cause fluid overload and precipitate congestive heart failure [21]. In case reports by Gautier et al. and Anand et al., IVIG has been even shown to trigger Takotsubo cardiomyopathy [22, 23]. Cardiac complications are also known with PLEX in patients with MG with or without Takotsubo [24]. Thus, careful monitoring of fluid status and cardiac function with either IVIG or PLEX is recommended. This is especially so in older patients and in those with severe myasthenia, as they are particularly susceptible to Takotsubo cardiomyopathy and the risk of associated congestive heart failure, prolonged hospitalization, and its associated complications with either rescue treatment [20].

## 4. Conclusion

MG patients may have other autoimmune disorders like Grave's disease, which can worsen neuromuscular junction dysfunction and precipitate crisis. Patients who develop Takotsubo stress cardiomyopathy during myasthenia crisis require early recognition of characteristic wall motion patterns and screening for other causes like hyperthyroidism to avoid prolonged hospitalization with increased morbidity and mortality. Meticulous cardiac and fluid monitoring especially in older patients and with severe myasthenia might be beneficial in these patients as they might be at increased risk for developing recurrent stress cardiomyopathy.

## Figures and Tables

**Table 1:** Cardiac abnormalities and thyroid function during two episodes of myasthenia gravis crisis.

MG crisis	TSH mcunit/ml	Peak troponin, ng/ml	ECG changes	Echo findings
Episode 1	7.6	0.16	ST elevation V1–V6, Q waves V1–V3	Mid ventricular variant Takotsubo stress cardiomyopathy with reduced ejection fraction (EF) of 25%

Episode 2	0.569	0.32	Deep, symmetric T inversions V1–V6	Mid and apical akinesia, EF 30% consistent with classical apical ballooning type of Takotsubo stress cardiomyopathy

## Abbreviations

MG: Myasthenia gravis
ECG: Electrocardiogram
TTE: Transthoracic echocardiogram
IVIG: Intravenous immunoglobulin
PLEX: Plasma exchange
LV: Left ventricle
TPO: Thyroid peroxidase.
